# Transient CFD analysis of fuel siphoning under a sudden inertial load

**DOI:** 10.1038/s41598-022-07572-y

**Published:** 2022-03-08

**Authors:** Kang-Sik Bae, Edwin L. Blosch

**Affiliations:** 1 Korea Aerospace Industries, Ltd., Sacheon, South Korea; 2 Computational Fluid Dynamics Specialist, Engineering and Technology, Lockheed Martin Aeronautics, Fort Worth, USA

**Keywords:** Aerospace engineering, Computational science

## Abstract

This paper presents the results of CFD investigations of siphoning between tanks of an aircraft fuel system under high-g conditions typical of an extreme pull-up maneuver. Negative gauge pressures in the siphon tank can occur, potentially breaking the siphon and decreasing the available fuel flow to the engine. A representative configuration of a system has been modeled, consisting of two fuel tanks plus a vent tank, fuel transfer lines, and gas lines for venting and supply of inert gas to the fuel tank ullage spaces. The fuel pressures and dynamics are simulated using a time-dependent Volume-of-Fluid modeling with normal force increasing rapidly from 1 to 9 g corresponding to an extended period of rapid pull-up during which the main supply tank becomes empty. Realistic values for engine fuel flow rate and fuel properties (JP-8) are used. This work is part of a broad effort to investigate fuel system performance issues that are difficult to test. Four simulations were performed in total, comparing 1 g flight and a 9 g pull-up both with and without a low-capacity pump assisting the siphon transfer. We find that the pump influences the siphon flow rate, the siphon break characteristics as the supply tank empties, and the magnitude of negative gauge pressures that occur in the siphon tank.

## Introduction

This paper presents the results of CFD simulations aimed at understanding potential worst-case negative pressures occurring in an aircraft fuel tank during a rapid pull-up (high g) maneuver. In particular, we are interested in the pressures that arise in a supply tank that delivers fuel to the engine but is itself supplied with fuel from another tank via a siphon line. This design is typical of a fighter aircraft fuel system. For the purposes of this study, a representative three-tank system is analyzed using CFD, comparing a baseline cruise operation against an extreme (9 g) pull-up maneuver.

The maneuver case is important from a design perspective. First, the combination of inertia and hydrostatic g loads is a limit case for the proper sizing of the fuel tank structure. Along with engine fuel flow requirements, this case must be considered in sizing the pumps and siphon lines in the system. The fuel system function can be impeded under high g loadings. The down force on the fuel in the siphon can break the siphon effect, reducing the transfer rate of fuel. Thus, it can be difficult to maintain the desired transfer rate of fuel to the engine.

There is limited information in the literature about the effect of negative gauge pressures on siphon performance in context of a real system layout. The present work provides insights and establishes a model that can be applied to consider other flight conditions and physical phenomena of real systems. Cavitation effects can be significant if fuel transfer rates are high^1^, and these are possible to include in CFD. In addition, outgassing of dissolved gas from the fuel^2^ can occur in rapid ascents, creating non-inert conditions.

The paper is organized as follows. First, a representative three-tank fuel system is described, which includes two low-capacity centrifugal fuel pump lines, a siphon transfer line, and vent and wash lines. Next, the CFD modeling is described. Then, simulation cases and conditions are presented. In the last section, the results of CFD-predicted transfer rates and pressures are summarized and discussed.

## Representative fuel system

**Figure 1:**
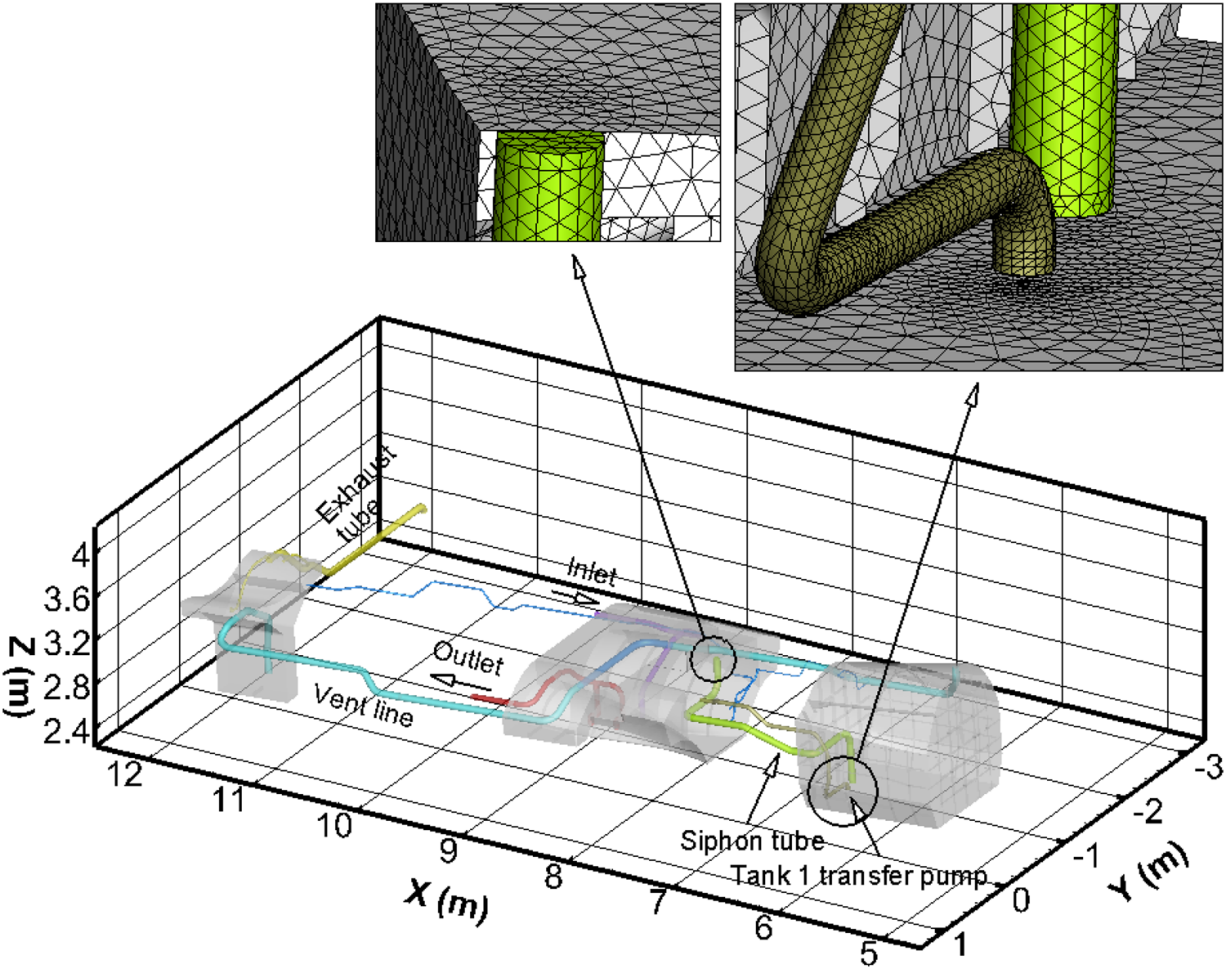
Isometric view of the three-tank representative fuel system.

The components and dimensions of the representative fuel systems are shown in Fig. 1. Three gray tanks are shown with transparency. The overall dimensions of the system can be seen, as well as several colored lines connecting the tanks, and close-up views of the CFD mesh at the intake and outlet of the siphon transfer duct between Tank 1 and 2. The system as a whole is oriented with the front end (Tank 1) slightly above the vent tank, according to the aircraft pitch attitude in a nominal cruise condition. Simulations are performed in a body-fixed coordinate system in which gravity acts in the negative Z direction; this is a worst-case combination of inertial and body-frame normal acceleration such as would occur in the bottoming out part of a dive and pull-up maneuver.

On the right is Tank 1. This is a supply tank which is emptied first as the engines consume fuel. Fuel in Tank 1 is pumped and siphoned to the middle tank, which is referred to as the siphon/Tank 2. Fuel is pumped out of Tank 2 to the aircraft engines. On the left is the vent tank, which opens to the external atmosphere via pressure relief valves under conditions of very high or very low pressures. In addition, the vent tank provides an overflow reservoir for spillage from Tank 1 when if it becomes too full, which can happen during some failure modes or during aerial refueling.

**Figure 2:**
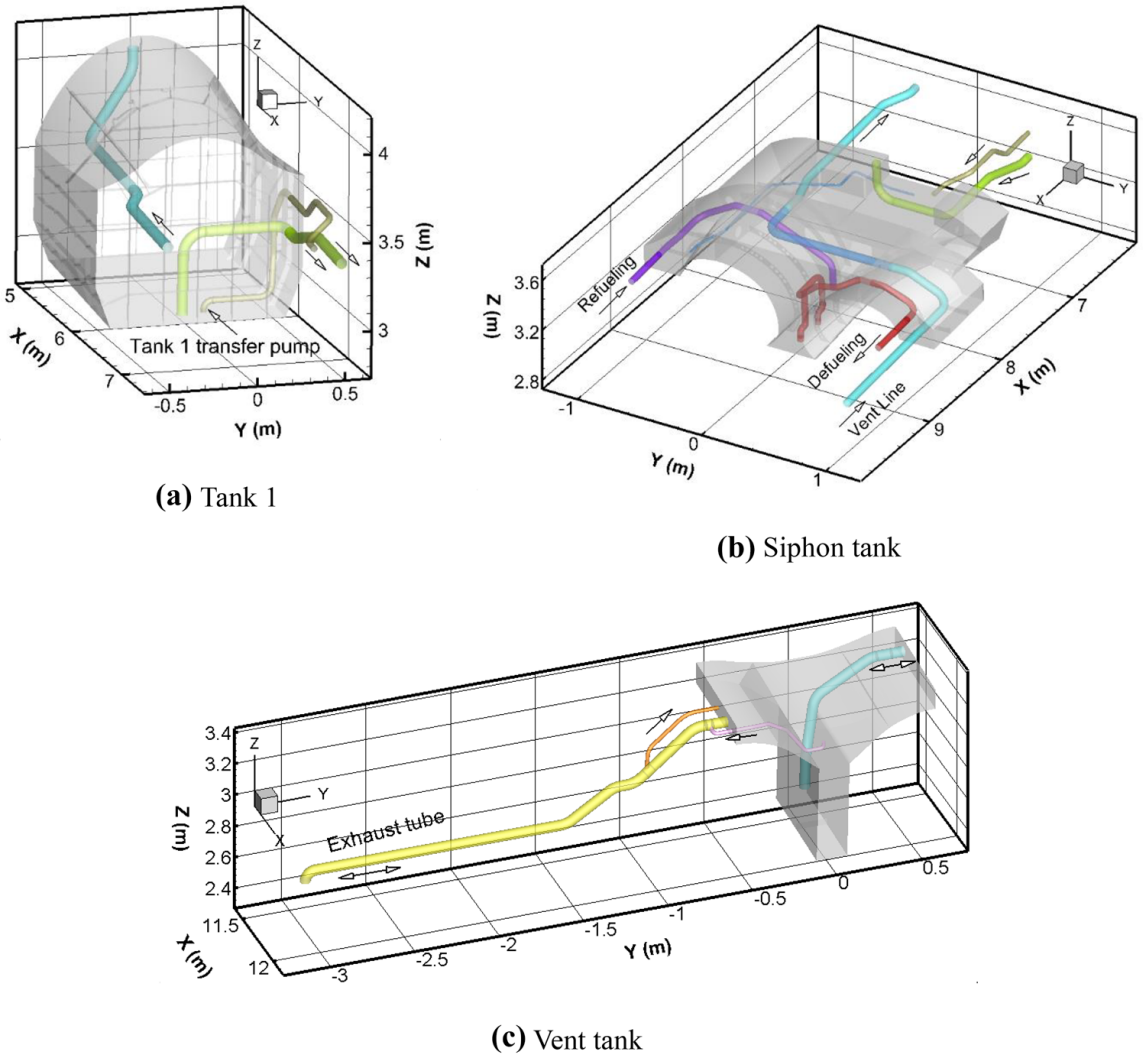
Closer views of the geometry of the three tanks.

Figure 2 shows close-up views of each of the three tanks. In Fig. 2(a), the cyan-colored line is a duct to the vent tank. One end is at the top corner of Tank 1, above the liquid fuel level. The other end is in the vent tank. The yellow-green line in Fig. 2(a) is the siphon transfer line. In Tank 1, the intake is at the very bottom of the tank, and the other end is at the very top of the siphon/Tank 2. The gold-brown line in Fig. 2(a) is the pump line from Tank 1 to Tank 2. A fan model is incorporated in the CFD model to pump the fuel through this line.

In Fig. 2(b), several lines are visible. The cyan line is the vent line (cyan), which in the case just passes through the siphon/Tank 2. The yellow-green line and gold-brown line are the siphon transfer and pump lines as previously described. The red line is the engine defueling line. It has two pumps, labeled forward and backward, and both are sometimes needed to meet the fuel demand, e.g. during usage of the afterburner. An afterburner is a thrust augmentation system in a gas-turbine engine system that involves injecting fuel downstream of the combustor and turbine to produce additional rocket-type thrust. The intakes to the engine defueling line are positioned at the bottom of the siphon/Tank 2 while the outlet is slightly above the mid-level of the tank. In the CFD simulations, a fixed mass-flow boundary condition is applied to the defueling line. The purple line in Fig. 2(b) is the refueling line. During the CFD simulations, the refueling line is closed using a mass-flow boundary condition and prescribed 0 kg/s flow rate.

In Fig. 2(c) the external exhaust line is colored yellow, the vent line to Tank 1 is colored cyan, and the purple and orange lines represent check and control valve lines, although the valves are not modeled in the present study (these lines are open). Not shown in Fig. 2(c) is the supply line coming into the vent tank from the OBIGGS system, which supplies nitrogen-enriched air (NEA) to keep the gases inert. This line can be seen in blue, however, in Fig. 1.

**Figure 3:**
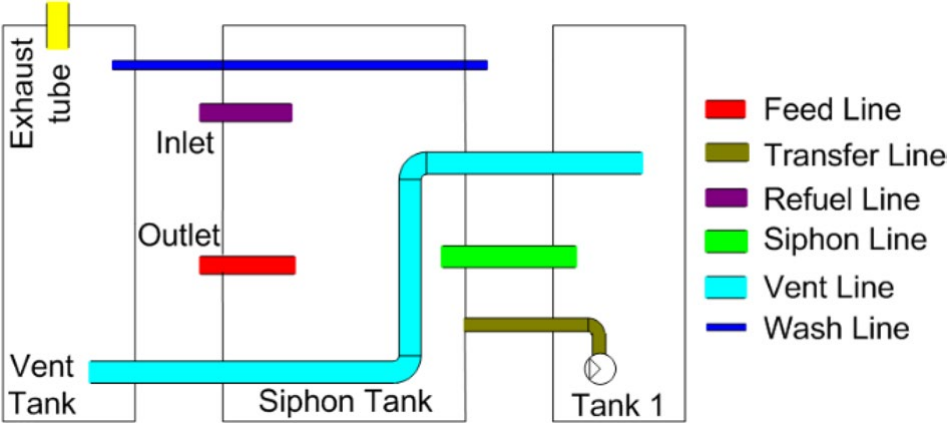
Top view schematic of the three-tank system.

Figure 3 shows a top-view logical schematic of all these connections. The most important ones for the present study are the siphon transfer line and pump transfer line. In typical systems, the siphon lines are large in order to reduce pressure drop and thus the pump power required to exceed the net positive suction head. In this system, though, the siphon line has a relatively large diameter (2.5 in.) in order to reduce the risk of accidentally breaking the siphon effect during hard maneuvers.

The system works as follows. During a normal cruise, Tank 1 transfer pump delivers fuel to the siphon/Tank 2 at a sufficient rate that the siphon line does not play a role. During an acceleration, as the defuel rate is increased out of the siphon/Tank 2, low pressure conditions are created, and this draws fuel from Tank 1 via the siphon line to augment the pump transfer. Peak demand establishes sizing. As Tank 1 empties, the intakes of both the siphon and pump lines become exposed, and pressures between the two tanks equilibrate. Generally, the fuel in the siphon line runs backwards into Tank 1 during this time, and the pump picks it up and continues pumping until Tank 1 is virtually empty. At that time, the Tank 1 transfer pump shuts itself off according to a sensor.

These dynamics are a strong function of the tank orientation and g loading, i.e. gravitational acceleration. The gravity vector is pointed in the negative body-Z direction in the present study. This is a conservative approximation that results in less than 1% error in modeling a dive and pull-out maneuver in which the gravity vector is canted 8 degrees forward of the body-Z axis at the beginning and 8 degrees backward at the end. This is sufficient for understanding the effect of the pull-up maneuver on the pressures in the tanks.

## CFD modeling approach

The representative fuel system was simulated using CFD with the two-phase, two-fluid volume-of-fluid (VOF) methodology, first proposed by Hirt and Nichols in 1981^3^. This modeling is appropriate because the liquid fuel and gas-phase ullage in the tank system are immiscible and remain mostly unmixed. The fuel in this work is considered to be JP-8, an incompressible liquid, and the gas is considered to be incompressible air. Details regarding the bulk fluid properties and the chemical composition of JP-8 can be found in^4^. In reality, the gas phase would be a mixture of air and fuel vapor, but the differences in viscosity and density are negligible for our purposes. The siphon tank ullage is modeled as incompressible flow because the ullage volume remains unchanged, which is because both transfer line and siphon line outlets are submerged in the fuel, inflows and outflows are balanced, the fuel itself is incompressible, and the tank volume is fixed, and the system is isothermal. The supply tank ullage is open to the external ambient conditions and inflow of air does not give rise to significant pressure variations.

The VOF method is built on the concept of a scalar phase fraction γ representing the volume of the CFD cell which is occupied by the primary phase. γ ranges as 0 ≤ γ ≤ 1, and values 0 and 1 correspond to cells in which only one phase is present. In this case, γ = 0 for the gas phase, and γ = 1 for fuel. Intermediate values correspond to cells which contain a fuel-gas interface. The phase fraction moves according to a simple advection transport equation for γ. Both phases share the same velocity field; this assumption is equivalent to a no-slip assumption at the free surface boundary. A concise explanation of the method can be found in Berberovic et al.^5^.

The governing equations that are solved in the VOF method are the equation of continuity, the transport of γ and the Navier–Stokes equation:

1 $$\nabla \cdot U=0$$

2 $$\frac{\partial \gamma }{\partial t}+ \nabla \cdot \left(U\gamma \right)= 0$$

3 $$\frac{\partial \left(\rho U\right)}{\partial t}+ \nabla \cdot \left(\rho UU\right)= -\nabla p+ \nabla \cdot \tau + \rho g+ \sigma \kappa \nabla \gamma $$

In these equations, $U$ represents the velocity field, which is considered to be the same for the two fluids throughout the flow domain. τ is the deviatoric viscous stress tensor, ρ is the phase-averaged density, *p* the pressure and *g* the gravitational acceleration. The density and viscosity are calculated as weighted averages based on the volume fraction, i.e.

4 $$\begin{aligned} &\uprho = {\rho }_{l}\gamma + {\rho }_{g}\left(1- \gamma \right)\\& \upmu = {\mu }_{l}\gamma + {\mu }_{g}\left(1- \gamma \right)\end{aligned}$$

where the subscript *l* denotes the liquid phase (fuel) and the subscript *g* the gas phase. In Eq. (3), σ is the surface tension coefficient and $\kappa$ is the local curvature of the free surface, and the last term represents the surface tension force as proposed by Brackbill et al.^6^. The curvature $\kappa$ is defined as using the phase fraction as follows:

5 $$\kappa = -\nabla \cdot \left(\frac{\nabla \gamma }{\left|\nabla \gamma \right|}\right)$$

The gradient of $\gamma$ is reconstructed for a given cell using a piecewise linear (geometric) approximation of the interface through the cell, considering its neighboring cells.

These governing equations are Favre-averaged to account for turbulent flow, in consideration of the Reynolds numbers estimated inside the siphon transfer duct and pump transfer lines. The flow speeds in the pump/siphon transfer lines are between 0.35 m/s and 6.9 m/s during the simulated maneuver and correspond to Reynolds numbers of 10,300 and 120,840 (based on pipe diameter). Laminar-turbulent transition in pipe flows occurs around 2,300, so we assume the flow is fully turbulent. The flow rates in the siphon/transfer lines depend on the net balance of hydrostatic pressure differences and pressure losses associated with the flow, hence the decision not to disregard the effects of turbulence.

The modeling of turbulent two-phase flows via RANS models in a VOF formulation is established practice^7–10^ and does not lead to any new turbulent-VOF fluxes as long as Favre-averaging is used^11,12^. Velocity gradients need some special care in discretization around interface cells, but this issue is the same as normally done for molecular viscous stresses. The usual caveat for RANS treatments applies, namely that if flows have large-scale unsteady turbulence characteristics, they will be better modeled by spatial-averaging techniques like LES. The key flows in our system are multi-phase turbulent pipe flows for which the RANS approximation is well-suited. Menter’s k – ω model with shear-stress transport (SST) was selected for turbulence modeling. In this model, two additional equations are solved for the transport of the turbulent kinetic energy k and the turbulent dissipation rate ω^13,14^, and wall function boundary conditions are automatically applied when y +  > 1. Ultimately the flow-driven pressure losses in this system are not large, so the sensitivity of results to the modeling of viscous effects was not evaluated.

The governing equations were discretized using second-order upwind discretization in space for the momentum equations, the Compressive scheme for the volume fraction (suitable for sharp interfaces between phases), and a second-order backward-differenced implicit formulation in time with variable time-stepping, for all equations. The time-step size change between steps was limited between 0.8 and 1.2. The discretized equations are solved using the PISO scheme^15^ in ANSYS Fluent version 17.1^16^.

Next we describe the computational mesh. An unstructured mesh generated using ANSA^17^ based on geometry imported from CATIA V6. The thickness of the transfer lines has been neglected in the geometry for convenience of meshing. The surface mesh uses triangular cells, and the volume mesh is comprised of tetrahedral cells. Best practices for the prediction of turbulent flows with wall function-supported turbulence models were followed. The predicted values of y+ (resolved Reynolds number based on wall spacing) were less than 50 everywhere. As discussed above, viscous losses are not the dominant physical consideration in modeling the development of negative pressures due to the maneuver. Thus, the primary concern in meshing was not high resolution of wall-bounded flows in the transfer lines, but rather the overall mesh quality and resolution of internal features of the geometry. The computational mesh is not refined enough to capture surface tension effects with high accuracy, but the refinement is sufficient for our purposes, because stratification and inertial effects are the dominant mechanisms.

As can be seen in Fig. 1, the mesh is refined in the region of the Tank 1 transfer pump inlet and the discharge connection of the siphon tube. The mesh resolution inside the pump transfer and siphon lines was uniformly 0.1 mm at the walls and increased to 4 mm at the centerline, less than 10% of the diameter of the siphon tube. A systematic mesh-resolution study was performed to assess the accuracy of this resolution for predicting the pressure drops in the pump and siphon transfer lines, which are the main factors in the transient evolution of siphon tank pressures and the siphon break event. Three successively finer meshes were created for the region surrounding these lines and their entrance/exit regions. Transient simulations were repeated for Case 3 (see Simulation Description below). The pressure drop through the siphon line in each case was compared at t = 50 s, which allows for potential accumulation of spatial and time-integration errors. We found that the solutions on each finer mesh approached an asymptotic value monotonically. Grid convergence was then assessed using the technique described by Roache^18^. The results are summarized in Table 1.

**Table 1:** Results of mesh-resolution study.

Grid	Resolution	Cell size (mm)	# Cells in siphon line	Pressure drop (kPa)	GCI	GCI ratio $\frac{1}{{2}^{\mathrm{p}}}\frac{{\mathrm{GCI}}_{2-3}}{{\mathrm{GCI}}_{1-2}}$
1	Fine	2.0	735,561	2769.3	0.31%	1.03
2	Medium	4.0	94,679	2688.8	4.06%	
3	Coarse	8.0	20,638	1672.2		

The grid-convergence index (GCI) is an estimate of the difference of a fine-grid solution from its value at infinite mesh resolution as determined by Richardson extrapolation of coarse and fine-grid solutions. The scaled ratio of successive GCI indices should approach 1 within the asymptotic range of mesh refinement; the value in our case is 1.03. This suggests that the results in this study, using the medium mesh resolution, should be within 4% of true values (using the safety factor of 3.0 as in^18^).

The mesh resolution decreases to 30 mm in the middle of Tank 2 (away from all surfaces), and the cell sizes transition smoothly between fine and coarse regions of the mesh. The maximum volume ratio between any two cells was 2.5 which is also considered best practice. The final mesh contained 2,094,344 cells. The quality of the mesh was high relative to typical experiences for complex geometries. The maximum cell skewness, which characterizes the shape of tetrahedral cells, was 0.87 on a scale of 0 (ideal) to 1 (bad). Skewness increases the distance between cell centers and faces, and thus high skewness decreases the accuracy of face-based flux terms. The minimum orthogonality was 0.103 on a scale of 0 (bad) to 1 (perfect). Orthogonality reflects how well-positioned a face centroid is between the two cell centroids on either side of it. This is a reflection of how accurately gradients across the face (viscous terms) can be evaluated.

## Simulation description

Four time-dependent simulations of defueling are performed. These are summarized in Table 2. All simulations were performed for 70 s total simulated time.

**Table 2:** List of cases simulated.

Case	Maneuver (70 s of simulation time)	Tank 1 transfer pump
1	1 g	OFF
2	1 g for 40 s, then transition to 9 g over 1 s, and hold	OFF
3	1 g	ON
4	1 g for 40 s, then transition to 9 g over 1 s, and hold	ON

**Figure 4:**
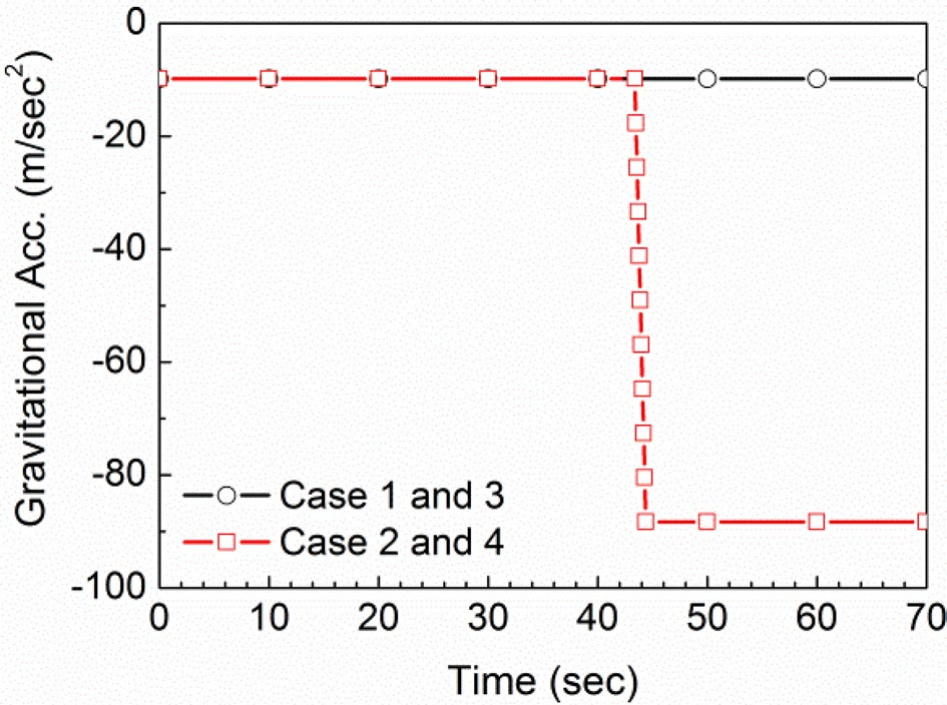
Time history of gravity acceleration.

The first case is a baseline no-maneuver case in which the pump transfer from Tank 1 to the siphon/Tank 2 is off, leaving only the siphon transfer. During this time, Tank 1 empties and the siphon tank remains full or nearly so, with the siphon tank defueling rate equal to the rate of fuel coming in from Tank 1. Case 2 is the same, except that at 40 s, there is a sudden pull-up to 9 g over 1 s, which is then sustained for the remainder of the time. This is shown in Fig. 4. The time-dependent gravitational acceleration was implemented in the Fluent CFD model using a user-defined function (UDF). The direction of the gravity vector corresponds to a 4° pitch attitude of the aircraft. It remains unchanged during the simulation time, which models the case of a pull-out maneuver level to the ground where inertial forces and body-Z axis acceleration are closely aligned. This gives a conservative result with respect to the negative pressures developed in the system.

Case 3 is the same as Case 1, a constant 1 g cruise baseline, except in Case 3, the Tank 1 transfer pump operates continuously. Likewise, Case 4 is the same as Case 2 (pull-up maneuver), except with the Tank 1 transfer pump on. The Tank 1 pump transfers fuel to the siphon/Tank 2. Thus, the transfer rates are higher in Cases 3 and 4 than in Cases 1 and 2.

The time-step size was automatically adjusted according to a specified global-minimum value of CFL number of 5.0, except during the transition period of pull-up from 1 to 9 g, during which the CFL number is reduced to 1.0. This typically produced values around 2.5 × 10^−4^ s and average CFL numbers throughout the domain much smaller than 1.0. In addition, ANSYS Fluent automatically sub-divides the time-step integration of the volume fraction to improve accuracy. Ten subiterations were used, and the continuity equation residual was reduced to 1 × 10^–5^ on each step to reduce the accumulation of numerical errors over the long-duration simulations. As a check on the validity of results, the difference between the final gas bubble separation value and the average value for the last five seconds of the flow time was less than 1%, which gives further confidence in the validity of results.

Simulations were performed on 200 cores using a 10-node, 20 cores-per-node Linux-based HPC cluster with 2.6 GHz Intel processors, and 16 GB of RAM per node. Total simulation times for the full 70 s duration were on the order of 10 days.

**Figure 5:**
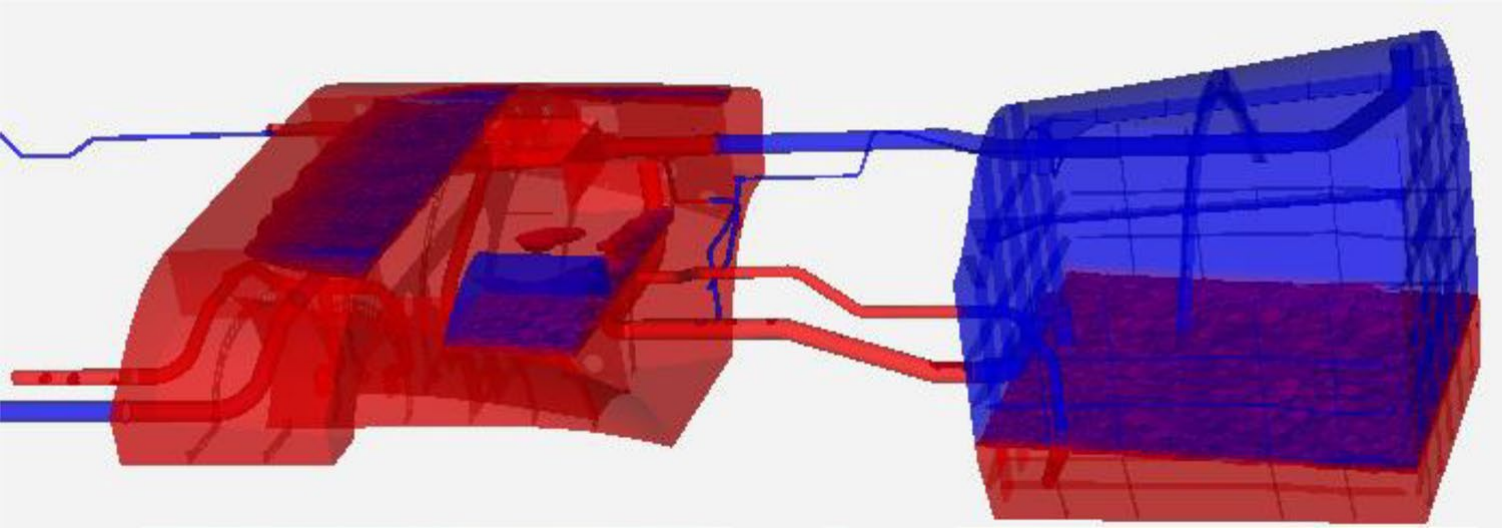
Initial conditions for all four defueling simulations.

The initial conditions (fuel levels) in Tank 1 and siphon/Tank 2 for these *defueling* simulations are established by a preliminary, partial *refueling* simulation using an inlet mass-flow-rate condition through the refuel line (see Fig. 1 and Fig. 2(b). The initial conditions correspond to a full siphon/Tank 2 and a 25% full Tank 1 by volume and are shown in Fig. 5 below. The fuel is red, and the gas is blue. The purpose of filling Tank 1 only 25% full was just to save computational time, as our interest is what happens with the siphon transfer as Tank 1 runs empty during a pull-up maneuver.

**Figure 6:**
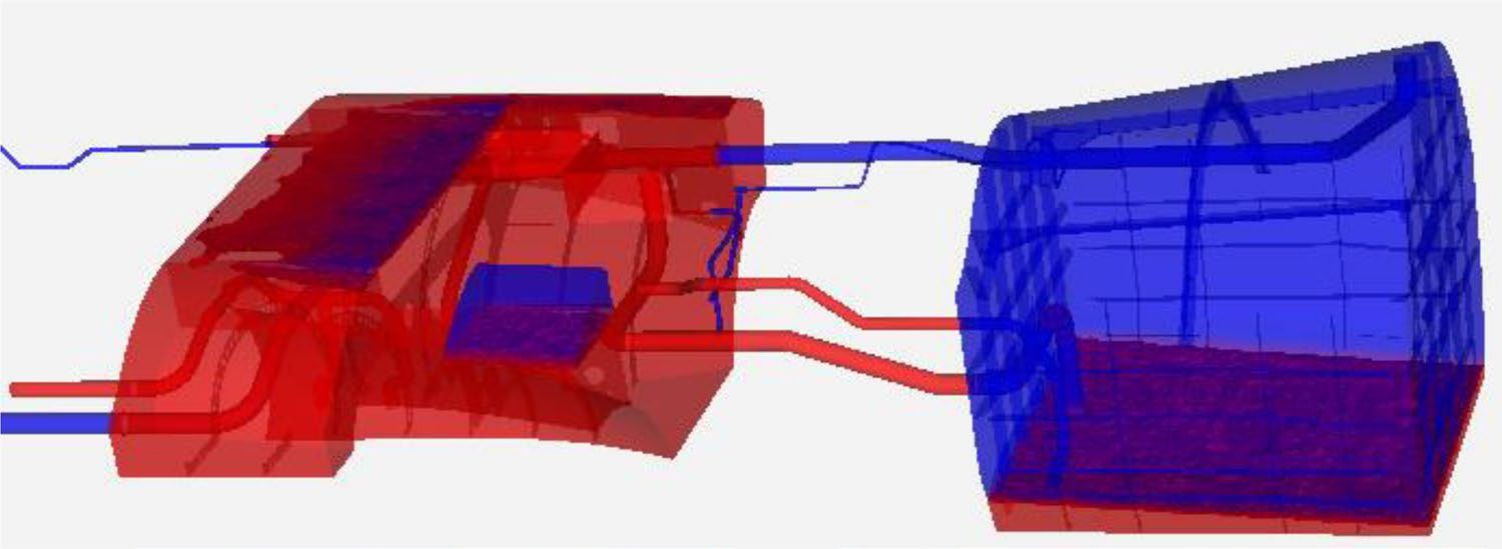
Intermediate restart result for Cases 3 and 4 at t = 40 s (beginning of pull-up).

For the first 40 s of simulation, Cases 1 and 2 are identical, as are Cases 3 and 4, because the acceleration to 9 g is not applied yet. To save computational time, the simulation for Case 2 was started as a restart from the t = 40 s result of Case 1. Likewise, Case 4 was a restart of from the t = 40 s result of Case 3. Figure 6 shows the “initial” conditions of Case 3 and 4 at the t = 40 s point. In effect, this is an intermediate result. It can be observed in Fig. 6 that Tank 1 is nearly emptied.

Inflow and outflow boundary conditions and the Tank 1 transfer pump model settings are summarized in Table 3. The Tank 1 transfer pump model is applied during Cases 3 and 4 only. During the preliminary simulation of refueling, approximately 5 kg/s fuel inflow is specified for the refuel line while the defuel line is set to 0 kg/s. These settings are reversed during the defueling simulation.

**Table 3:** Inflow/Outflow Boundary and Source Conditions.

Boundary/source	CFD specification	Refuel simulation	Defuel simulations
Fuel inlet to Tank 2 (Refuel line)	Specified mass-flow inlet	5.0896 kg/s	0 kg/s
Fuel exit from Tank 2 (Defuel line)	Specified mass-flow inlet w/outward-directed normal	0 kg/s	4.7292 kg/s
Vent exhaust	Specified pressure inlet/outlet	1 atm	1 atm
NEA supply line	Specified mass-flow inlet	0 kg/s	0.001889 kg/s
Tank 1 transfer pump (Cases 3 and 4 only)	3D fan model	Not applied	Pump speed: 523.5 rad/sec Pressure jump: 48,263 Pa

The material properties of JP-8^4,19^ and air used in the present work are shown in Table 4. All values are taken to be at 15.5 °C.

**Table 4:** Fluid properties.

	Air	JP-8
Molecular weight, MW (g/mol)	28.966	173
Density, *ρ* (kg/m3)	1.225	806.7
Dynamic viscosity, *μ* (mPa s)	0.017894	1.742
Surface tension, *σ* (mN/m)	-	23
Vapor pressure, p_*v*_ (Pa)	-	244.6

## Results and discussion

The CFD simulations were set up to calculate and monitor mass flow rates and pressures at selected points throughout the simulations. Pressures were monitored at the entrance and exit of the Tank 1-to-Tank 2 siphon transfer line, and the entrance and exit of the Tank 1-to-Tank 2 pump line. Mass flow rates were also recorded for these lines. The fuel volume in each tank was also monitored. Finally, internal surface pressures on the tank walls and volume fraction contours were examined through visualization to gain additional insight.

### Defueling process

**Figure 7:**
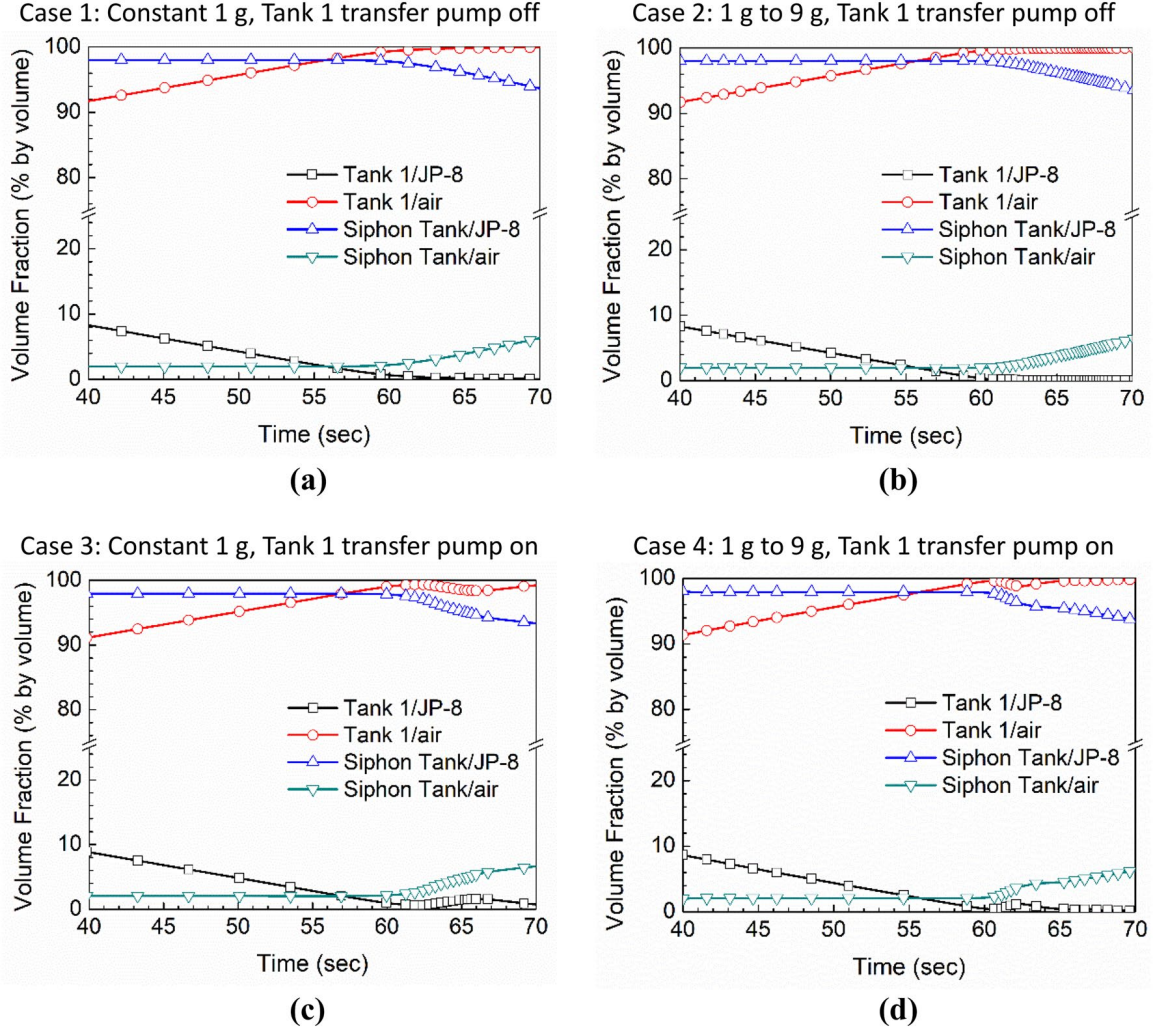
Volume fractions of JP-8 and air for each of the cases.

Figure 7 presents the fuel volume in the two tanks as a function of time. Fuel inside the siphon transfer and pump transfer lines is not counted as part of either tank’s fuel volume. Only the period from 40 to 70 s is shown. Prior to 40 s, the Tank 1 fuel volume (black line) is simply decreasing at a linear rate that matches the constant defueling rate, starting from its initial condition (25% full tank). The siphon/Tank 2 fuel volume is not changing at all (it remains 98% full), because the fuel flows in and out are exactly balanced.

In Fig. 7(a) and (b), the Tank 1 transfer pump is off, and thus the fuel transfer from Tank 1 to Tank 2 is performed completely by the siphon transfer line. In Fig. 7(c) and (d), both the siphon transfer line and pump are delivering fuel to the Tank 2. However, because the defueling rate is the same in all cases, the differences in the fuel transfer technique are not evident in these plots until approximately 60 s, when the Tank 1 volume reaches zero (empty).

At that time, differences appear between the siphon-only cases, Fig. 7(a) and (b), and the siphon-plus-pump cases. As Tank 1 runs empty, the siphon effect is broken by entrained air, and the fuel in the siphon line flows back into Tank 1, increasing its volume slightly (black line). The pump picks up this fuel and transfers it to the Tank 2, and the fuel volume returns to zero. Meanwhile, because Tank 1 is now empty, the Tank 2 starts to empty (blue line) at the specified defueling rate.

**Figure 8:**
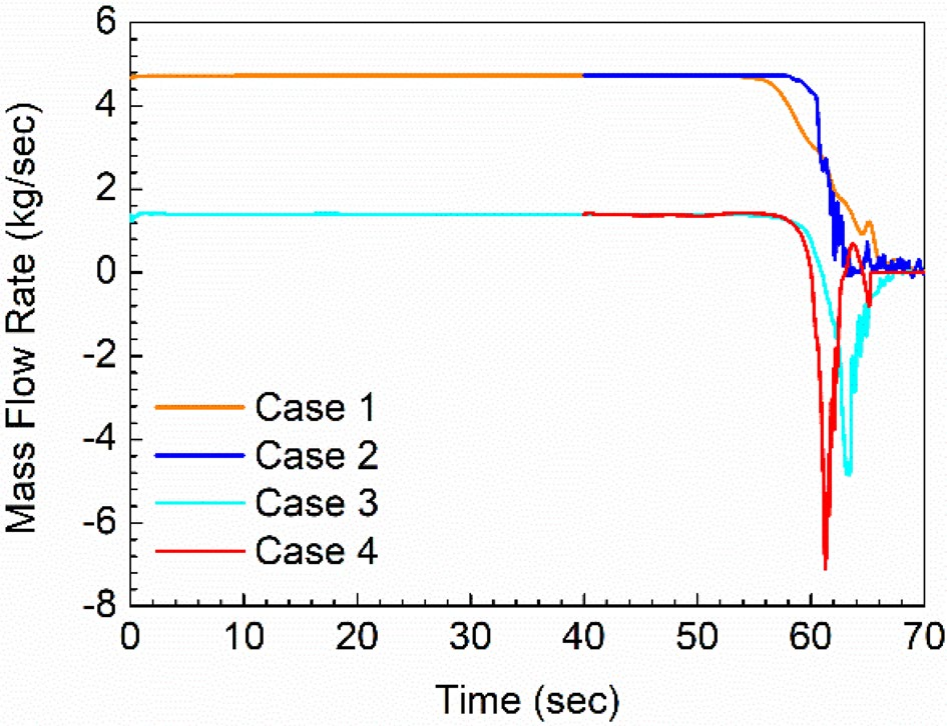
Fuel mass flow rate through the siphon tube.

Figure 8 shows the mass flow rate through the siphon line for each of the four cases. Positive values indicate flow into the siphon/Tank 2 from Tank 1. This data was extracted from CFD by integration over the cross-section of the siphon line near its discharge location in the siphon/Tank 2.

In the cases where the Tank 1 transfer pump is off, Cases 1 and 2, all the fuel transfers via the siphon line, matching the specified defueling rate of 4.7292 kg/s. The siphon break clearly occurs earlier in Case 1 (1 g) than in Case 2 (9 g). More air is required to enter the line before the siphon effect can be broken in the case where the suction pressure is higher (Case 2). In both Case 1 and 2, the siphon transfer rate is always positive; Tank 1 drains nonlinearly but continuously, until it is empty.

Cases 3 and 4, with the Tank 1 transfer pump on, behave differently. In both these cases, the siphon transfer only comprises 30% of the total, about 1.4 kg/s. We observe that the siphon break occurs at the same time in both cases, and that the fuel in the siphon line flows back into Tank 1 as the siphon is broken (negative flow rate). Physically, the siphon breaking process involves the rapidly pressure increase in the siphon/Tank 2 ullage to match the Tank 1 air pressure as air mostly fills the siphon line and, without a pressure differential, gravity causes the fuel to flow back into Tank 1. This process is faster in Case 4 because the weight of the fuel is much greater than Case 3 (9 g, vs. 1 g). The Tank 1 transfer pump is still pumping, so this fuel is quickly transferred to Tank 2 by the pump; see Fig. 7.

### Siphon/Tank 2 negative gauge pressures

**Figure 9:**
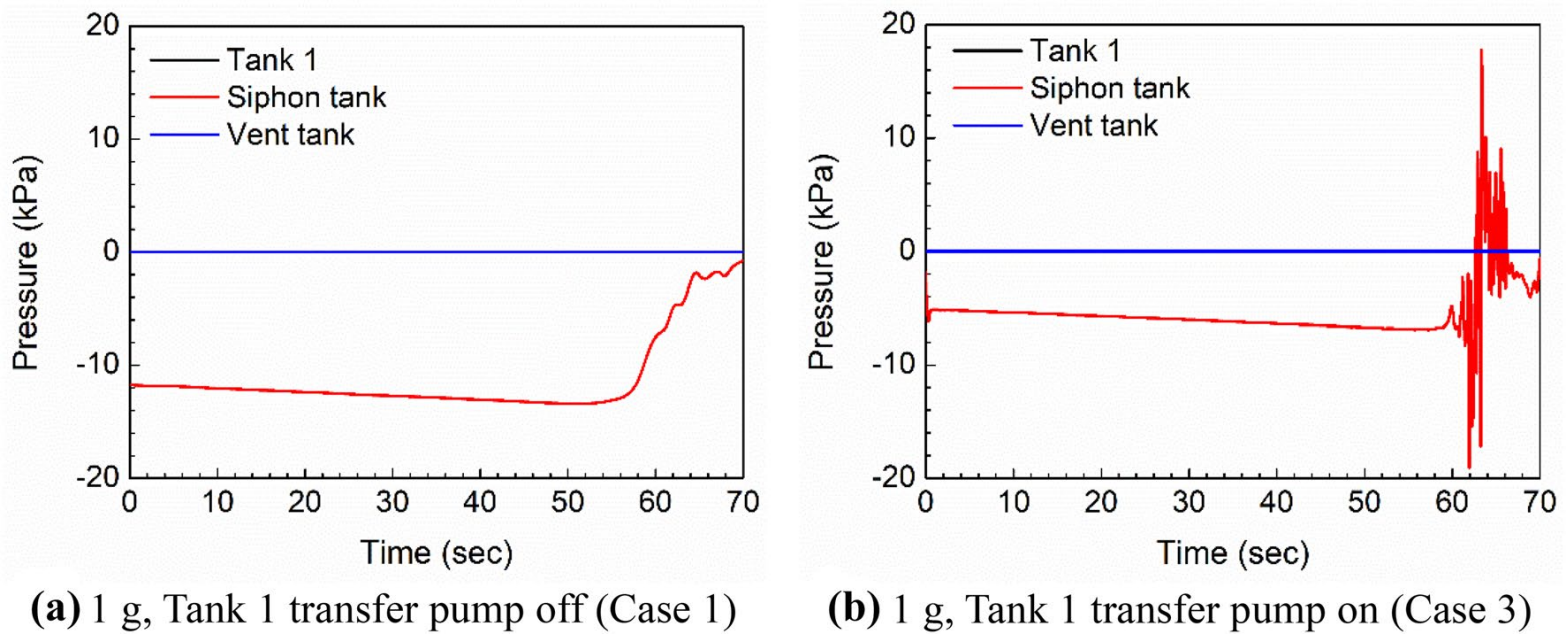
Comparison of siphon tank pressure in the 1 g case for the full 70 s duration.

Additional insight comes from examining the pressure in the siphon/Tank 2. Figure 9 presents these results for the 1 g cases (Cases 1 and 3). Note that the Tank 1 ullage pressure and vent tank pressure are effectively the same and equal to the external pressure, so the black and blue lines overlap each other and are zero. The Tank 2 ullage pressure is measured near the outlet of the siphon line. By design, Tank 2 remains full while Tank 1 empties, so that the outlet end of the siphon line remains under the fuel level until the Tank 2 fuel level begins to decrease, creating an ullage space at the end of the siphon line.

The negative gauge pressure that develops in the siphon tank is a function of the pressure differential required to meet the defueling rate, which depends on the pressure differential between Tank 1 and Tank 2. In the case of the siphon-only transfer, Fig. 9(a), a differential of about − 12 kPa is developed to overcome the pressure head difference between the tanks, and the pressure losses in the siphon tube. In the siphon-plus-pump case, the CFD simulations predicted that the pump would transfer 63% of the total mass flow rate. As shown in Fig. 9(b), this reduces the negative pressure required in the siphon tank to about − 6 kPa. As time progresses, Tank 1 empties, and so the pressure head between the tanks increases, and the pressure in the siphon tank decreases slightly. This continues until air starts to enter the siphon tube around 60 s.

The siphon break occurs slightly earlier in Case 1 than Case 3, Fig. 9(a) vs. Fig. 9(b). This appears to be related to higher inlet velocities (increased separations) at the intake of the siphon tube. As air starts to enter to the pipe, the frictional pressure drop decreases and finally there is only air in the tube, and we see the pressure in the siphon tank recovering almost to the same level as Tank 1. In this final condition, the mass flow of air from Tank 1 to Tank 2 is approximately 0.0004 kg/s. The pressure in the Tank 2/siphon tank ullage in Fig. 9(b) is much noisier. This is caused by the location of the pressure measurement being intermittently in the fuel and above the fuel during the siphon breaking process. As the siphon transfer is suddenly disrupted and the pump transfer surges in response, the fuel–air interface sloshes around, causing this noise at the pressure measurement location. At certain moments, the siphon/Tank 2 gauge pressure is greater than the Tank 1 ullage pressure.

**Figure 10:**
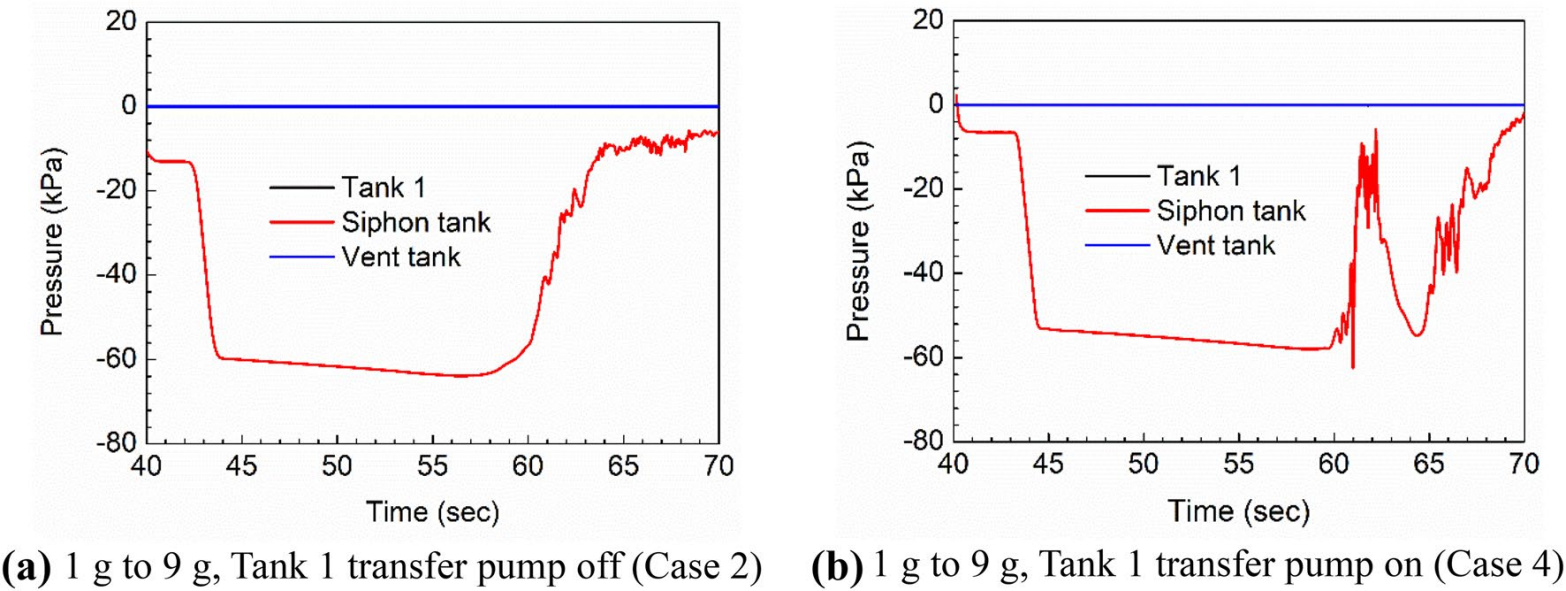
Comparison of siphon tank pressure in the 1 g to 9 g maneuver case.

Figure 10 shows the same results for Cases 2 and 4, with the 9 g loading initiated from 43.5 s and 44.5 s, to contrast with Fig. 9. Note that the x-axis scale is different between Figs. 9 and 10. Only the data after 40 s is plotted, because the first 40 s are identical to Fig. 9. Extreme negative pressures occur during the maneuver and are sustained until the siphon break occurs. Extreme values are considered design-critical loads for structural design. Figure 10(a) shows that the siphon tank ullage pressure recovers to the 1 g levels after the siphon break. In Fig. 10(b), as with Fig. 9(b), the measurement point is again sometimes in the fuel and sometimes in the ullage, but the pressures in the fuel are more negative due to the 9 g loading, so there is no moment when the siphon tank pressure exceeds the Tank 1 ullage pressure.

The ability to assess conditions that may break the siphon is of great interest to mission planners because, if the siphon transfer is interrupted suddenly, the fuel flow to the engine would be suddenly decreased, resulting in a reduction of aircraft speed and maneuverability. As a follow-up step, we assessed our simulation results to see if the siphon break could be well-described by a typical empirical parameter (denoted σ) found in the description of cavitation of valves^20^, defined for the purposes of this study as:

6 $$\upsigma = \frac{({P}_{1}- {P}_{v})}{({P}_{1}- {P}_{2})}$$

Here, ${P}_{1}$ is the pressure inside the siphon line near its entrance in Tank 1, ${P}_{2}$ is the downstream pressure inside the siphon line near its outlet, and ${P}_{v}$ is the vapor pressure of JP-8 at the system temperature, which in our case is 244.677 Pa^4^.

In the description of cavitation in valves, $\upsigma$ sometimes correlates well to the onset of cavitation and beginning of damage associated with the energy released by collapsing bubbles^21^. Although the siphon break process is not associated with cavitation in the present simulations, a real fuel system does have check and control valves which may experience cavitation. Also, although the current model which does not model any valves, the $\upsigma$ parameter may still correlate to siphon-break onset, because the overall mechanism is the same, i.e. air gets into the line and reduces the pressure differential that maintains the suction. In real fuel systems, the siphon effect can also be broken due to sloshing or pressurization effects.

**Figure 11:**
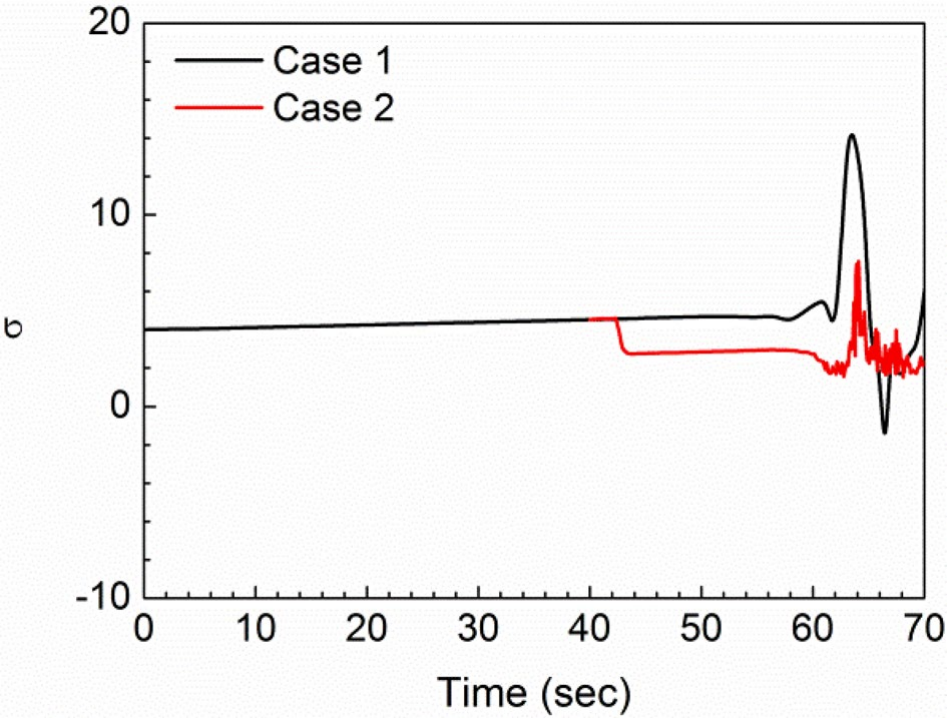
Cavitation parameter $\upsigma$ for Tank 1 transfer pump off cases (black = 1 g, red = 1 g to 9 g).

Figure 11 compares σ for the two cases with the Tank 1 transfer pump off, i.e. siphon-only transfer.

In both cases, the evidence of a sudden positive spike in the cavitation parameter at the point of siphon break is evident, followed by a small positive or negative final value. The 9 g case (red line) also shows that $\upsigma$ is closer to zero in this case, which reflects a greater potential for siphon break. In general, this parameter must remain positive and large to avoid cavitation at surfaces and solid impurities in the fuel, and to avoid excessively low dynamic pressures. It appears that the parameter does show a useful sensitivity, but further study is needed to determine meaningful threshold levels for the present system.

### Fuel tank surface pressures during 9 g maneuver

**Figure 12:**
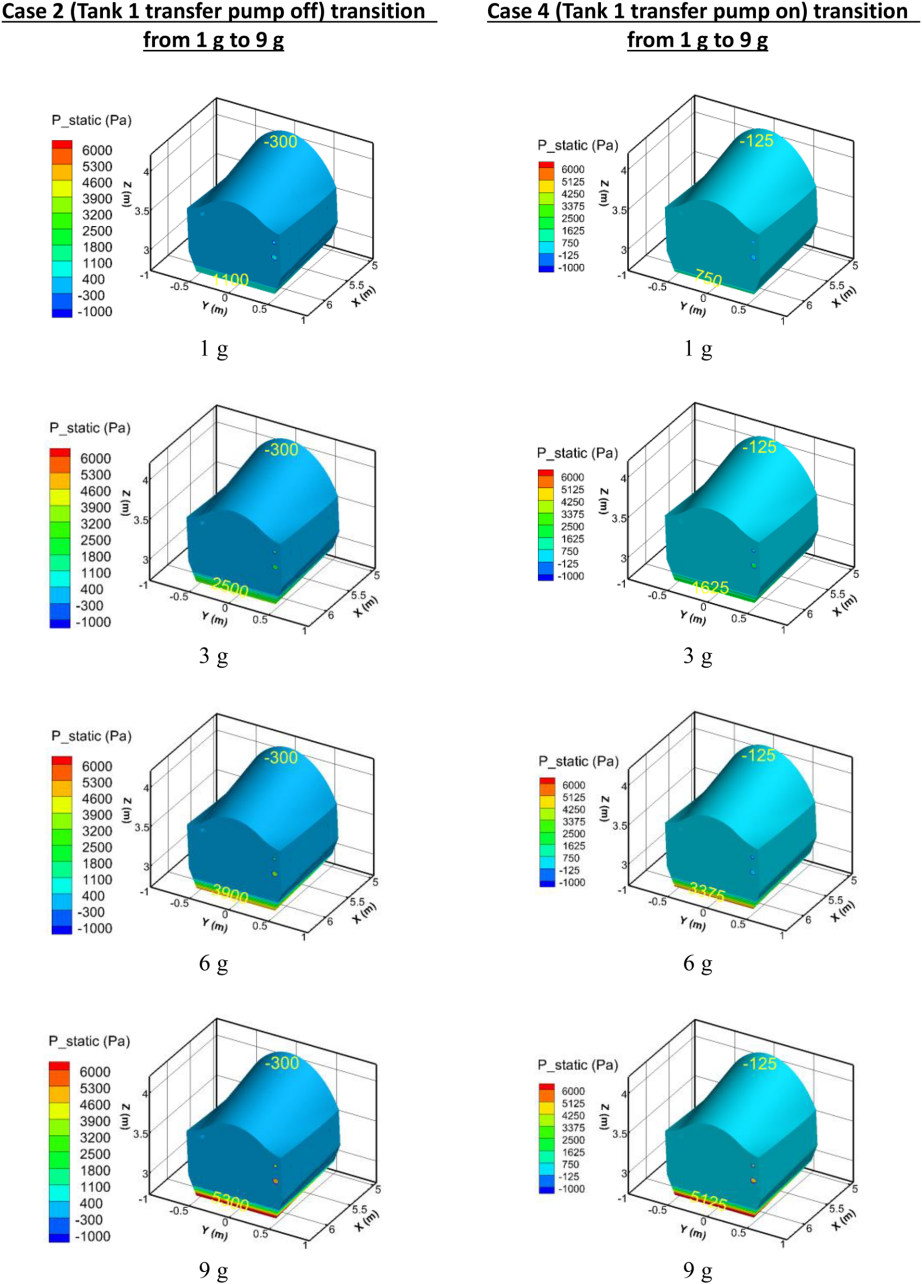
Pressures in Tank 1 during acceleration to 9 g.

Figure 12 shows the surface pressures that arise in Tank 1 at instants during the loading to 9 g, while Table 5 summarizes the differential between top and bottom. Recall that Tank 1 is less than 25% full at the moment when sudden acceleration occurs. On the left side the plots correspond to Case 2 (Tank 1 transfer pump off, siphon-transfer only), while on the right side the plots are for Case 4 (siphon and pump both transferring fuel).

**Table 5:** Tank 1 Pressure differential during acceleration to 9 g.

Gravity	p_bottom_—p_top_ (Case 2) (Pa)	p_bottom_—p_top_ (Case 4) (Pa)	Undershoot (%)
1 g	1400	875	38
3 g	2800	1750	38
6 g	4200	3500	17
9 g	5600	5250	6

The effect of the pump is evident in these results. Because the pump offsets the pressure head that would otherwise be required to deliver fuel to the siphon/Tank 2, the pressure differential in Tank 1 is decreased by 38% over what it is without the pump. However, at 9 g, the pump is doing relatively less work compared to the siphon transfer, so the Tank 1 pressure differential is only 6%.

**Figure 13:**
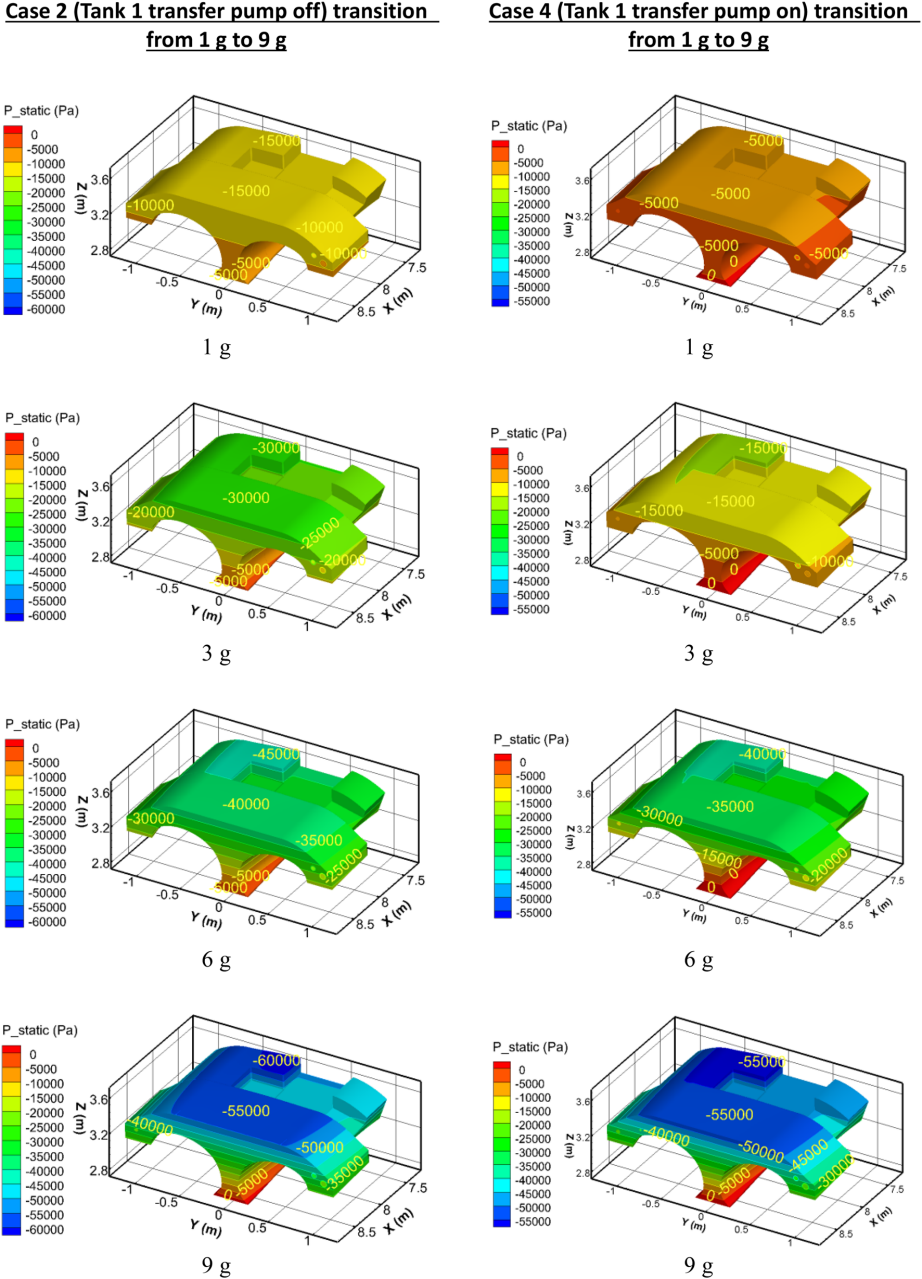
Pressures in the siphon/Tank 2 during acceleration to 9 g.

Figure 13 shows the pressure on the tank surfaces of the siphon/Tank 2 at the same instants of acceleration from 1 to 9 g loading as in Fig. 11. Table 6 summarizes the top wall pressures that occur.

**Table 6:** Siphon/Tank 2 Top Wall Pressures.

Gravity	p_top_ (Case 2) (Pa)	p_top_ (Case 4) (Pa)	Undershoot (%)
1 g	− 15,000	− 5000	67
3 g	− 30,000	− 15,000	50
6 g	− 45,000	− 40,000	11
9 g	− 60,000	− 55,000	8

It is evident that the pump is doing most of the work at the 1 g level, while at the 9 g level, the siphon tube is doing the majority of the work. The pump is helping to reduce the negative pressures by about 8%. The bottom wall pressures in Fig. 13 show values around -5 kPa, but the top wall has an extreme value of − 60 kPa.

## Conclusions

The present work has explored two-phase VOF simulations on a simplified, representative fuel system to gain insight into the effect of a high g maneuver on siphon transfer performance and fuel tank system pressures. The CFD modeling was able to predict the extreme negative pressures that are expected in the siphon tank under such maneuvers, as well as the changes in system pressures that occur as Tank 1 empties. The siphon break event was also predicted, and the simulations even predicted the flow-back of fuel into Tank 1 in the case where the Tank 1 transfer pump was on (Case 4). The results show that the siphon breaks at the same time with or without g loading, but the presence of extreme loading changes the work-share of fuel being transferred by the pump and siphon lines, and the siphon break duration is decreased. In addition, the pump reduces the peak suction pressures by almost 10% in the siphon tank. One can infer from these results that designers would want to include the beneficial effect of critical loads reductions in selecting pump and siphon line diameters.

The Volume of Fluid (VOF) method was surprisingly robust and produced physically realistic results, despite the complexity of the geometry and simulations. This may be because the flow is mainly stratified, and the inertial effects are dominant over the interface forces. The fuel and air are also both incompressible, and this approximation along with small time-step sizes helped ensure that non-physical mass accumulations or losses did not occur over the duration of the simulated time.

Based on the success of the current study, future efforts will be undertaken to extend this model to include wing tanks and more realistic Tank 1 transfer pump behavior. In reality, the Tank 1 transfer pump turns off after a few seconds after sensing that the fuel is depleted. This is a relatively easy realism to add into the CFD model. The further developed model will then be used to examine additional time-dependent maneuvers, varying pitch attitude of the aircraft, and sloshing loads.
